# RE-AIM Planning and Evaluation Framework: Adapting to New Science and Practice With a 20-Year Review

**DOI:** 10.3389/fpubh.2019.00064

**Published:** 2019-03-29

**Authors:** Russell E. Glasgow, Samantha M. Harden, Bridget Gaglio, Borsika Rabin, Matthew Lee Smith, Gwenndolyn C. Porter, Marcia G. Ory, Paul A. Estabrooks

**Affiliations:** ^1^Dissemination and Implementation Science Program of ACCORDS, Department of Family Medicine, School of Medicine, University of Colorado, Aurora, CO, United States; ^2^Physical Activity Research and Community Implementation, Human Nutrition, Foods, and Exercise, Virginia Tech, Blacksburg, VA, United States; ^3^Patient-Centered Outcomes Research Institute, Washington, DC, United States; ^4^Department of Family Medicine and Public Health, School of Medicine, University of California, San Diego, La Jolla, CA, United States; ^5^Center for Population Health and Aging, Texas A&M University, College Station, TX, United States; ^6^Department of Environmental and Occupational Health, School of Public Health, Texas A&M University, College Station, TX, United States; ^7^Department of Health Promotion and Behavior, College of Public Health, The University of Georgia, Athens, GA, United States; ^8^Department of Health Promotion, College of Public Health, University of Nebraska Medical Center, Omaha, NE, United States

**Keywords:** RE-AIM, evaluation, external validity, dissemination, implementation

## Abstract

The RE-AIM planning and evaluation framework was conceptualized two decades ago. As one of the most frequently applied implementation frameworks, RE-AIM has now been cited in over 2,800 publications. This paper describes the application and evolution of RE-AIM as well as lessons learned from its use. RE-AIM has been applied most often in public health and health behavior change research, but increasingly in more diverse content areas and within clinical, community, and corporate settings. We discuss challenges of using RE-AIM while encouraging a more pragmatic use of key dimensions rather than comprehensive applications of all elements. Current foci of RE-AIM include increasing the emphasis on cost and adaptations to programs and expanding the use of qualitative methods to understand “how” and “why” results came about. The framework will continue to evolve to focus on contextual and explanatory factors related to RE-AIM outcomes, package RE-AIM for use by non-researchers, and integrate RE-AIM with other pragmatic and reporting frameworks.

## Introduction

The RE-AIM framework (1) was developed to address the issue that the translation of scientific advances into practice, and especially into public health impact and policy, have been slow and inequitable (2–6). RE-AIM and other models (7) have helped balance the traditional focus on internal over external validity. Unique features of RE-AIM include an explicit focus on issues, dimensions, and steps in the design, dissemination, and implementation process that can either facilitate or impede success in achieving broad and equitable population-based impact.

The seminal RE-AIM paper (1) has been cited over 2,800 times, and the RE-AIM framework has been applied to study planning or evaluation in over 450 publications (7). RE-AIM is one of the most frequently used frameworks for planning and evaluation of grant applications at most of the leading U.S. health and medical research agencies (8) and has been used widely (nationally and internationally) (9, 10) and across populations, settings, and health conditions (11–24). Generally, RE-AIM does seem to translate and be useful in the different countries and cultures in which use has been reported. Some international applications include low- and middle-income countries (25) including Australia (26–30), the Netherlands (31–34), and Brazil (35, 36). One interesting application was the use of RE-AIM to help plan and evaluate interventions to reduce the use of coal-fired indoor cook stoves in Africa (37). In this article, we summarize the history of the RE-AIM framework, discusses current applications of RE-AIM for research and practice, and outline opportunities for future application.

## Historical Perspectives

The dimensions of the RE-AIM framework were originally introduced to encourage scientists to be more transparent and consider internal and external validity across pilot, efficacy, effectiveness, demonstration, and translational research (23, 38). Most peer reviewed publications previously emphasized efficacy, leaving researchers, and practitioners with little information about the generalizability of the intervention context, implementation personnel and conditions, and findings. The main goal from its conception was to improve assessment and reporting along the five RE-AIM dimensions, not necessarily intervening to improve all dimensions (see Table 1).

**Table 1:** The RE-AIM dimensions: definitions, evolution, and examples from the literature.

**Dimension**	**Definition**	**Historical perspectives**	**Current issues and outcomes**	**Future directions**
**Reach** Click here for more information	The absolute number, proportion, and representativeness of individuals who are willing to participate in a given initiative, intervention, or program. Reasons for not participating Click here for information on improving reach, such as: “*How do I reach the targeted population with the intervention?”*	- Reporting on demographic characteristics - Comparison between participants in different study conditions and between those who stayed in the intervention and those lost to follow-up - Unknown as to the degree to which those in the intervention were similar to the target audience	- Description of target audience (including a best estimate denominator) - Comparison of sample to the target audience (representativeness) - Use of a number of factors to best calculate the proportion reached (39) - Some use of qualitative methods to understand “why and how”	- Use of reach implementation strategies to improve access, awareness, and appropriateness of intervention to meet the target audience needs - More focus on recruitment strategies (and interventions) to directly address health equity (2) - Reach as an outcome target for dissemination trials.
Examples from the Literature	- Worksite wellness intervention started with a brief health survey of all participating worksites; participants were not informed that there may be a future worksite intervention. Results indicated that, once offered the worksite wellness intervention, “employees from higher income households, with higher education levels and health literacy proficiency were significantly more likely to participate in the program (p's < 0.01)” (40). - Community health promotion intervention for African American and Hispanic or Latina women. Investigators found that African Americans were more likely to not meet eligibility criteria and that the Hispanic/Latina women were more likely to drop out. There were no significant differences by city or recruitment method. In addition, at the end of the study participants “overrepresented higher educated, wealthier, and older women” (41).			
**Effectiveness** Click here for more information.	The impact of an intervention on important outcomes, including potential negative effects, quality of life, and economic outcomes. Heterogeneity of effects and reasons for success or lack of such Click here for information on improving effectiveness, such as: “*How do I know my intervention is effective?”*	- Reported subjective or objective measure related to the primary outcome (e.g., change in diet, smoking cessation, physical activity behavior, or biomarker such as hemoglobin A1c) - Exclusive focus on average overall effect and often one single outcome	- Still reporting primary outcomes; - Some studies are also measuring quality of life (QOL) and unintended consequences (42) - More emphasis on subgroup effects	- Need greater attention to QOL, unintended consequences, and systems impacts - For those participants who do experience an unintended consequence, more information on proposed “next steps.” - Relationships among multiple outcomes and relationship of context to RE-AIM outcomes
Examples from the Literature	• In a community adaptation of a trial, body image satisfaction was measured as a secondary outcome of a child's weight loss intervention. Almost half of the overweight children [*n* = 16 of the 34 (47%)] exhibited a decrease in body dissatisfaction at 6 months compared with baseline (43). However, five children (15%) had an increase in body image dissatisfaction. • Diabetes self-management support web assisted program effectiveness outcomes included improvements in quality of life, but no unintended negative consequences were measured (44).			
**Adoption** Click here for more information.	The absolute number, proportion, and representativeness of: a) settings; and b) intervention agents (people who deliver the program) who are willing to initiate a program. Reasons for adoption or non-adoption Click here for information on improving adoption, such as: “*How do I develop organizational support to deliver my intervention?”*	- Limited to no information on rates and representativeness of *staff* and settings that participate - Reporting only on these settings and staff who participate	- More studies reporting setting level adoption rates - Few studies reporting representativeness at the setting level - Few reporting on multi-level adoption issues - Somewhat greater use of qualitative measures	- Need to better understand contextual factors related to adoption - Need more information on multiple setting level characteristics [e.g., organizational culture and climate (45)] - Development of guides and tools to help users enhance adoption (and other RE-AIM outcomes)
Examples from the Literature	• Full RE-AIM evaluation of a 10 week school-based nutrition education program for third graders. Adoption was measured at the third-grade classroom level. Thirty-nine percent of all third-grade classrooms across all public schools in the targeted state participated. No information on representativeness of the schools that did or did not participate (46). • Print materials tailored for Korean American women: adoption was a secondary outcome and interviews were used for adoption level data. Qualitative adoption results included that the print materials were easy to include and that this contributed to adoption (47).			
**Implementation** Click here for more information.	At the setting level, implementation refers to the intervention agents' fidelity to the various elements of an intervention's protocol, including consistency of delivery as intended and the time required. Also includes adaptations made and the costs of implementation. At the individual level, implementation refers to clients' use of the intervention and implementation strategies. Click here for information on improving implementation, such as: “*How do I ensure the intervention is delivered properly?”*	- Limited or no information on time, costs and resources needed to complete intervention components well and over-time. - Only fidelity reported, never adaptations	- Increased attention to strategies to improve implementation of an intervention - Improvements on standardized measures for capturing implementation fidelity. - Much recent attention to adaptations - Limited links of implementation quality, adaptations and impacts to other RE-AIM outcomes	- Need greater uptake of implementation measurement protocols (48) - Multi-method assessments of implementation and adaptation - Multi-level and practical assessments of costs and combining implementation cost with proportion of participants benefiting from intervention - More understanding of reasons for adaptations and high/low levels of implementation - Rapid, iterative use of RE-AIM assessments to guide adaptations
Examples from the Literature	• A pragmatic, mixed-methods, quasi-experimental study across five community hospitals. Three hospitals received the nurse-administered Tobacco Tactics intervention and two received usual care. Intervention was streamlined, user friendly, etc. and resulted in nurses increased provision of advice to quit, counseling, medications, handouts, and DVD (all *p* < 0.05) when compared to control (49). • A community-based implementation trial of a cancer educational intervention was offered to 14 African American churches. Community health advisors were trained in a Traditional classroom setting or via the Web. Implementation outcomes included adherence, dosage, and quality. Implementation was strong across both conditions (all churches fully completing the workshops), but Traditional churches took more time to complete the workshops than the Web-based group. Notably, “other implementation outcomes were comparable between both the Traditional and Technology groups (*p* > 0.05),” which showed promise for using “web-based methods to disseminate and implement evidence-based interventions in faith-based settings” (50). • A community-wide, technology-facilitated weight-loss program was implemented in Colorado and reached over 30,000 overweight or obese community residents. Implementation costs were derived using payer invoices and combined with the reach (number of participants) and effectiveness (proportion of participants to achieve a 5% weight loss) to determine cost per participant with a clinically meaningful weight loss. Costs varied based upon participant characteristics (representativeness) in that African American participants saw a lower cost per clinically meaningful weight loss due to a higher retention and success rate while costs per participant remained relatively constant (51).			
**Maintenance** (individual and organizational) Click here for more information.	The extent to which: a) behavior is sustained 6 months or more after treatment or intervention; and b) a program or policy becomes institutionalized or part of the routine organizational practices and policies. Includes proportion and representativeness of settings that continue the intervention and reasons for maintenance, discontinuance or adaptation Click here for information on improving maintenance, such as “*How do I incorporate the intervention so that it is delivered over the long term?”*	- Long term outcomes seldom reported - RE-AIM somewhat arbitrarily selected 6 months post intervention as default (1) - Ongoing challenge of relapse after intervention is withdrawn - Previous helicopter research: Unknown system-level impacts beyond the study lifespan	- Limited data on outcomes post intervention (with no intervention contact) - High attrition from post program to 6 month follow up unless there are intervention “contacts” - Most maintenance data reported relate to other dimensions - For example, those who *maintained* the behavior were more likely to exhibit certain characteristics - Improvements in collaborating with end-users to enhance intervention fit and sustainability - Proportion of settings still delivering intervention remains the most commonly reported metric within this dimension (52)	- Ongoing intervention is often needed to sustain impact - Need strategies for relapse prevention within large-scale interventions [-] Greater understanding of factors leading to sustainment - Partnership with intended delivery system is ubiquitous with successful institutionalization (53) - Need pragmatic measures and systems-level buy in to ensure that relevant data are collected beyond the “research” phase (54, 55) - Need more understanding of dynamic, complex multi-level factors related to sustainment
Examples from the Literature	• *Se*tting level: To reduce depression outcomes in primary care, a collaborative-care management strategy called Community Based Outpatient Clinics (CBOCs) was deployed in the Department of Veterans Affairs. Eleven sites engaged in the study, and once funds were withdrawn, 91.9% (10/11) continued to apply the CBOCs approach (56). • *Se*tting level: Evaluation of continued implementation of a new computer-based intervention tool for lifestyle intervention in primary health care, 2 years after its introduction. Clinics either had explicit (e.g., theory-based training and support) or implicit (e.g., non-theory-based introduction with no ongoing support) strategies for tool use. Units with explicit strategies were more successful at the onset of the intervention, but over 24 months, those effects were mitigated (57).			

The RE-AIM dimensions include reach (R), effectiveness (E), and maintenance (M)–which operate at the individual-level (i.e., those who are intended to benefit), and adoption (A), implementation (I), and maintenance (M), which focus on the staff and setting levels. Setting-level RE-AIM factors are often multi-level and address context and external validity issues important to population impact. For example, settings may include clinics, schools, or worksites nested within communities or larger systems, and within these settings are nested clinicians, teachers, or human resources staff responsible for implementation.

All RE-AIM dimensions are complex, but implementation currently has the most indices. It focuses on fidelity to an intervention: the extent to which the program is implemented consistently across different settings, staff, and patients. It also includes adaptations made (58) and costs from multiple stakeholder perspectives (59). Maintenance has indices at the individual- (long-term effectiveness) and setting-level (sustainability after original research funded is completed).

The framework's operational components have been increasingly applied over the years. For example, in the past, studies reported participant characteristics that differed between study conditions or between those retained and those lost to follow-up. However, studies using RE-AIM compared the representativeness of individuals who enrolled in a study to the characteristics of the intended population. These comparisons used in RE-AIM studies increased understanding about access, awareness, appropriateness, and likely generalizability of recruitment strategies and intervention approaches.

In the past, clinical effectiveness research focused relatively narrowly on physiologic outcomes. RE-AIM expanded this focus to multiple factors that impact *public health*. This approach to assessing broader impacts aided in understanding comprehensive effects of a program on quality of life, including unintended consequences (e.g., increasing health inequity or the social stigma of labeling someone with a chronic condition).

There have been several literature reviews on use of RE-AIM (11, 42, 52, 60–62). The most comprehensive reviews have spanned the literature from 2000 to 2012 or 2015 (10, 42, 52). Notably, these reviews of different content areas reached similar conclusions: that adoption and maintenance, as well as representativeness across individual- and organizational-levels, were reported far less frequently. They identified frequent issues with confusing different dimensions, in particular reach (at the individual-level) and adoption (at the setting-level). These observations are not limited to the United States alone.

To enhance development and application of the framework, several scientists contributed to a RE-AIM research consortium funded by the Robert Wood Johnson Foundation (63–66). This work led to the development of a website, www.re-aim.org, in 2004 (64). The website serves as a repository of various resources and tools including self-quizzes, checklists, figures, tables, measures, tips for using RE-AIM, and increasingly, other social media tools. These are available to facilitate the operationalization and application of RE-AIM across diverse interventions, settings, and populations. To enhance a dialogue within the broader research community, monthly webinars are held about RE-AIM related issues; archived recordings are available on the website (www.re-aim.org).

## From Past to Present

Below, we summarize five general areas currently being examined using RE-AIM (Table 1). The first is to understand and maximize the potential of RE-AIM to assess adaptations prior to, during, and after program implementation (54, 55, 67). Adaptations naturally occur during the implementation of programs (68). Mittman et al. (69) suggest that instead of ignoring or suppressing this phenomenon, we should find ways to document and assess these changes. Recent Patient-Centered Outcomes Research Institute (PCORI) Methodology Standards (70) suggest that adaptations should be systematically documented. RE-AIM has great potential to provide guidance about documenting adaptations. It can also provide guidance about how to evaluate the impact of these adaptations, as well as their purpose (58). RE-AIM has been used to expand the widely known Stirman framework (71) on adaptations with additional components to address “who, what, why, where, and when” questions (67). RE-AIM considers adaptations in a longitudinal, multi-method, and multi-level manner and includes data collection at multiple time points and from multiple stakeholders, using multiple data collection approaches (54, 55, 67).

Second, there has been a recent focus on more qualitative RE-AIM assessments. Most evaluations and uses of the framework have emphasized descriptive or quantitative data, often focusing on key aspects such as the percentage of potentially eligible persons or settings that participate. A qualitative focus as presented by Holtrop et al. (72) can enhance understanding about what happened as well as the “how” and the “why.”

Third, we recognized the need for more pragmatic uses of the framework rather than trying to comprehensively assess all RE-AIM dimensions in all applications, especially when not having many evaluation resources (67, 73). All studies or evaluations, and particularly those without large evaluation budgets, do not need to assess all components of RE-AIM. Rather, they should address those components most valued and appropriate for their particular question, setting, stakeholders, and stage of research. An *a priori* decision should be made, however, to select the dimensions on which to focus for evaluation and on which to use for planning and improvement (i.e., beyond the evaluation scope). In some cases, the decision to capture all five dimensions is made *a priori* to understand individual impacts, contextual implications, and feasibility of ongoing data collection. This is demonstrated in two recent applications of RE-AIM before, during, and after program implementation—and on limited funds (74, 75). Both applications highlighted the need for stakeholder buy-in (54) and operationalization (67) of each dimension that holds value for these stakeholders.

**Figure 1:**
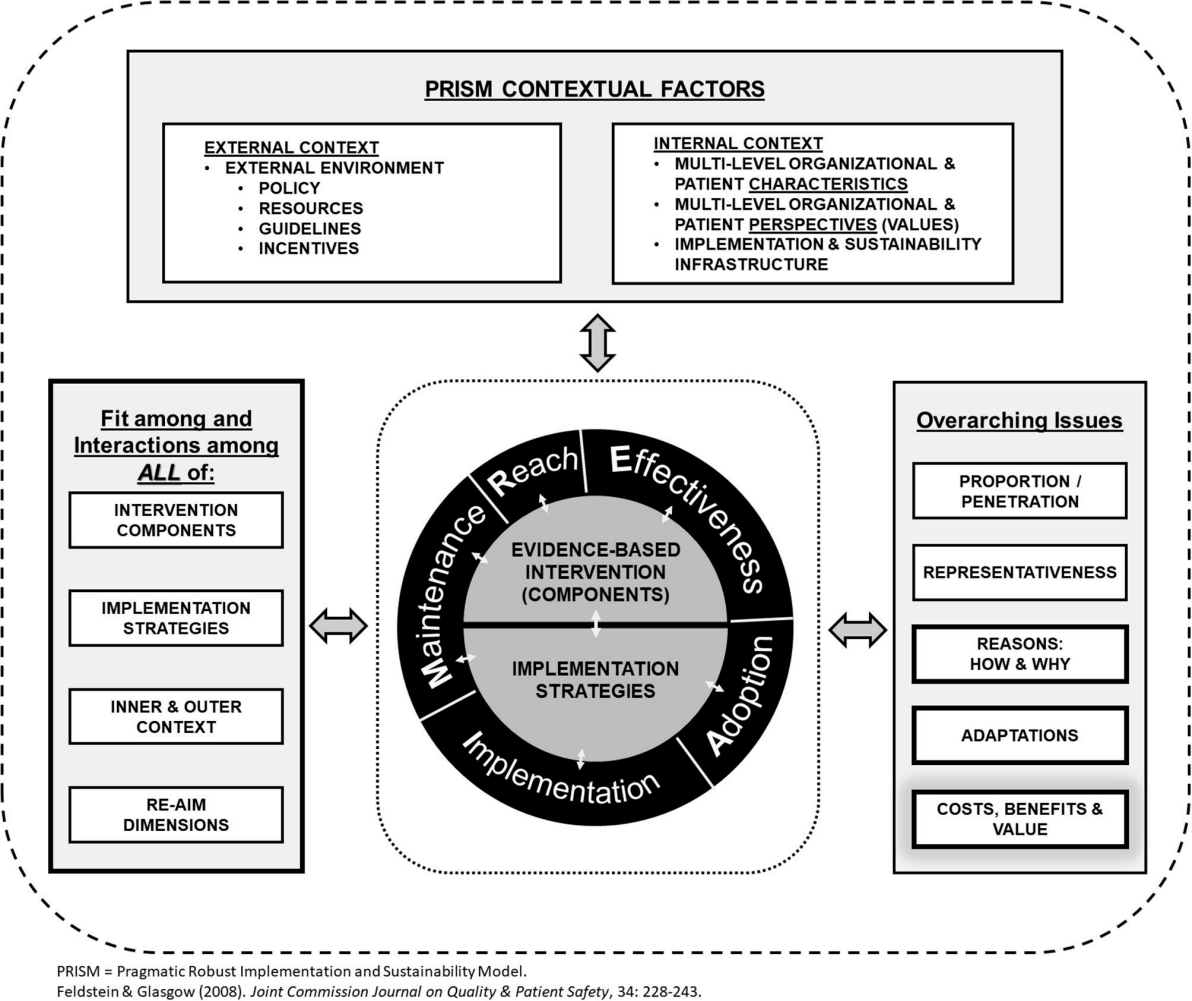
Revised, enhanced RE-AIM/PRISM 2019 model.

Fourth, assessment of costs, from the perspective of multiple stakeholders and across the various RE-AIM dimensions, is another area of emphasis (5). Building upon earlier work by Ritzwoller et al. (76), recent RE-AIM cost assessments have focused on the multilevel nature of implementation, different stakeholder perspectives, and cost estimates for replicating a program or policy in different settings. Rhodes et al. (59) have provided templates to assess costs at the patient-, staff-, clinic-, and organizational-levels. Costs to deliver programs are associated with activities to address and enhance each RE-AIM dimension (Figure 1). In the future, we anticipate more consistent reporting of costs and burden and more frequent comparative effectiveness research about cost-effective methods to enhance value and various RE-AIM dimensions.

Finally, Glasgow et al. (77) have recently advocated for an extension of RE-AIM concepts and dimensions, termed an Expanded CONSORT Figure to enhance transparent reporting, and potentially, replication. The goal is to expand the CONSORT reporting criteria required for randomized studies (78) to (a) include factors related to setting and staff level participation and representativeness, which begin before individual participants are recruited, and (b) extend the temporal focus beyond the end of a study. The expanded CONSORT figure and a related downloadable template summarize issues of exclusion and inclusion criteria for settings (e.g., communities or healthcare networks) and delivery staff, [e.g., evaluating the percent and characteristics of settings and staff that participate or do not (adoption)], reasons for participation or non-participation, and intervention sustainability after project support ends (79).

Based on these observations, we have developed a new RE-AIM figure to highlight the various changes to the model, as well as new emphases, including explicit inclusion of costs and adaptations, as shown in Figure 1. The figure also emphasizes key multi-level contextual factors (both the internal and external context) that influence RE-AIM outcomes as discussed below. Two crosscutting issues are: (a) that it is critical that there is alignment across setting and context, the intervention and implementation strategies; and (b) it is important to include qualitative assessments to determine how and why various RE-AIM outcomes are produced.

## What Will the Future Bring?

With the historical context and current application of RE-AIM in mind, we outline five future directions for researchers and practitioners interested in using RE-AIM.

First, there have been recent calls to more explicitly describe strategies and context (80, 81) as well as test mediating relationships between implementation strategies and implementation outcomes (82). We also see this as an emerging area for RE-AIM. The most well-articulated attempt to do this so far is the Practical, Robust, Implementation, and Sustainability Model (PRISM) (83, 84) that focuses on specific contextual factors from external macro-level factors such as policies, guidelines, and incentives, to more local organizational-level factors. It focuses on the fit between the characteristics of an intervention (i.e., Rogers' constructs of relative advantage, complexity, compatibility, observability, trialability, and cost) (85) and the particular intervention and implementation system. A somewhat unique factor of PRISM is its focus on enhancing setting-level maintenance characteristics by addressing the “implementation and sustainability infrastructure”—including job requirements, ongoing audit and feedback, and institutionalization of intervention activities (84, 86).

Second, mixed-methods should be used across framework components to identify explanatory processes across RE-AIM dimensions. To date, quantitative measures alone have been insufficient to strongly predict dissemination (reach and adoption), implementation, and maintenance outcomes. Using mixed-methods approaches can help identify factors that are causally related to different RE-AIM outcomes in different situations (72). Qualitative information integrated with newer predictive modeling approaches should provide more detailed guidance about actions that can be taken to enhance outcomes by addressing empirically-derived causal relationships.

Third, we encourage more iterative applications of RE-AIM and use of the framework during the implementation period, not just for initial planning and summative evaluation. Rapid, iterative use and analysis of brief practical measures of RE-AIM factors can inform adaptations (55, 66, 71). In brief, RE-AIM can be used as part of a participatory approach (Estabrooks et al., under review), to determine which dimensions should be assessed, described, or targeted for intervention. For example, a recruitment strategy may need to be adapted over the life-course of an intervention (to improve reach) or a new training strategy may be employed (to improve adoption and implementation). While often examined and interpreted independently, these adaptations can work together to be empirically robust and practically meaningful.

Another issue to be addressed is use of RE-AIM by non-researchers and groups such as state health programs or program evaluators without substantial funds (grants/contracts). Using RE-AIM in low-resource and real-world settings can be challenging but successful (2). Preliminary findings assessing such use are that RE-AIM is used widely, and seems to be relatively intuitive, but there are challenges implementing it at a detailed level and assessing all components. The development of user-friendly tools and aids using human centered design, as well as more examples of the application of RE-AIM for such users is an important future direction.

Finally, we think there is great opportunity for RE-AIM to be used in combination with other approaches such as the Pragmatic Explanatory Continuum Indicator Summary (PRECIS) model (87, 88), where RE-AIM factors can be combined with the PRECIS-2 dimensions to determine how pragmatic a study is and how generalizable it is likely to be. Such use is illustrated in a recent systematic review by Luoma et al. (89), who demonstrated how reviews can simultaneously summarize effectiveness (using Cochrane-type criteria) and pragmatism (using a combination of PRECIS-2 and RE-AIM factors). RE-AIM and its Expanded CONSORT extension could also be integrated with the Standards for Reporting Implementation Studies (StaRI) (90) or other dissemination and implementation (D&I) research reporting criteria.

## Conclusion

RE-AIM has been applied in research and practice for 20 years. Although its original components have remained, much has been modified and evolved to address emerging issues such as adaptation and dissemination costs. We expect that RE-AIM will continue to evolve to better address and enhance its original purpose—to increase the prevalence of relevant research that can be applied broadly across a wide variety of populations and settings to achieve a large, equitable, and replicable public health impact. We invite researchers and practitioners to contribute to the expanded use of RE-AIM before, during, and after intervention delivery.

## Author Contributions

All authors listed have made a substantial, direct and intellectual contribution to the work, and approved it for publication.

### Conflict of Interest Statement

The authors declare that the research was conducted in the absence of any commercial or financial relationships that could be construed as a potential conflict of interest.
